# Supplementary material for: Extensive iron–water exchange at Earth’s core–mantle boundary can explain seismic anomalies
Source: Nat Commun. 2024 Oct 15;15:8701. doi: 10.1038/s41467-024-52677-9 (PMC11480218; doi:10.1038/s41467-024-52677-9)
Supplement: Supplementary file 1 — Supplementary Information [file 41467_2024_52677_MOESM1_ESM.pdf]

Supplementary Information for "Extensive iron–water exchange at Earth's core–mantle boundary explains seismic anomalies"

Katsutoshi Kawano[1], Masayuki Nishi[1,2*], Hideharu Kuwahara[2], Sho Kakizawa[3], Toru Inoue[4], Tadashi Kondo[1]

[1] *Department of Earth and Space Science, Osaka University, 1-1 Machikaneyama-cho, Toyonaka, Osaka 560-0043, Japan.*

[2] *Geodynamics Research Center, Ehime University, 2-5 Bunkyo-cho, Matsuyama, Ehime 790-8577, Japan.*

[3] *Japan Synchrotron Radiation Research Institute, 1-1-1 Kouto, Sayo-gun, Hyogo 679-5198, Japan.*

[4] *Department of Earth and Planetary Systems Science, Hiroshima University, 1-3-1, Kagamiyama, Higashi-Hiroshima, Hiroshima 739-8526, Japan.*

*Corresponding author

Masayuki Nishi (nishimasa@ess.sci.osaka-u.ac.jp)

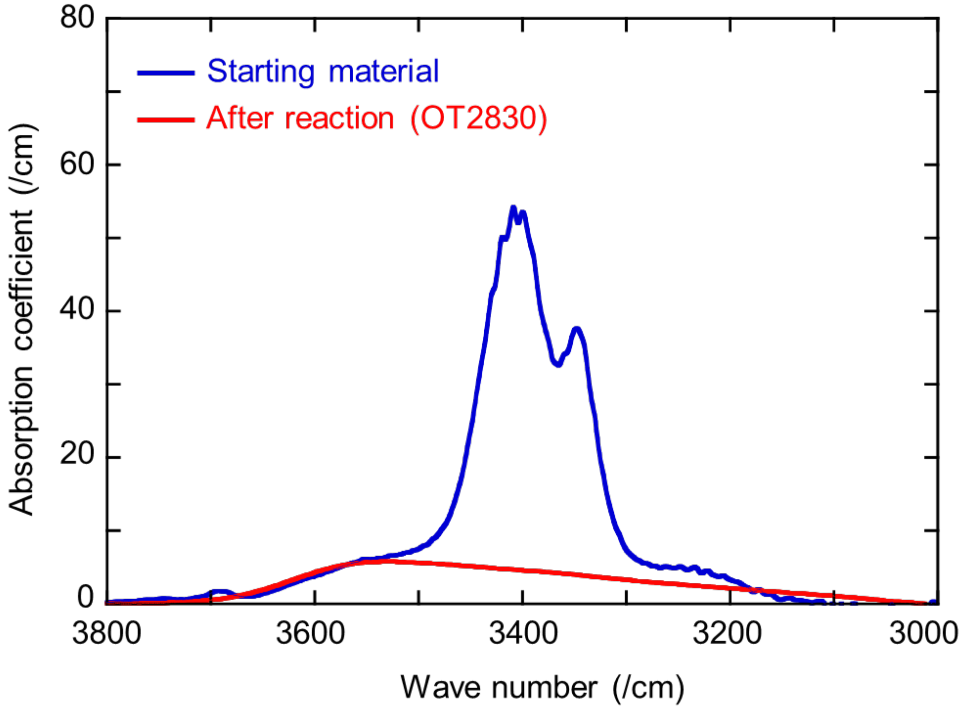

Supplementary Figure 1: FTIR spectra of bridgmanite. Blue and red solid lines indicate the FTIR spectra of the initial bridgmanite and that after iron–water exchange, respectively. Absorptions that originated from OH stretching vibrations were considerably reduced because of iron–water exchange.

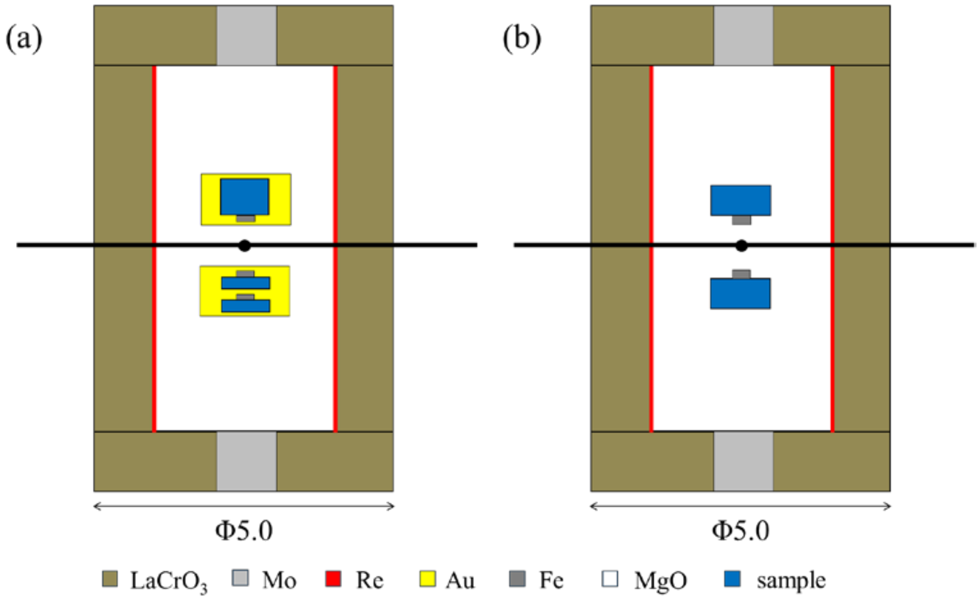

Supplementary Figure 2: Cross-section of sample assemblages. a, lower-temperature experiments. **b.** higher-temperature experiments.

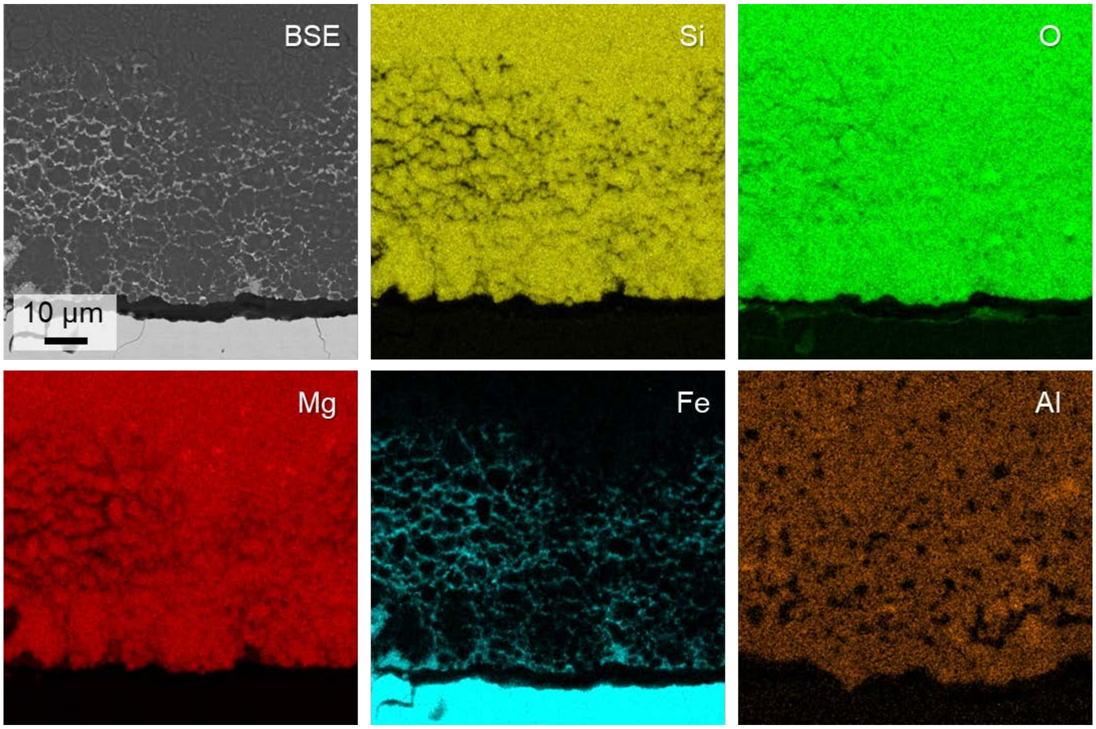

Supplementary Figure 3: BSE image and EDS elemental maps of run product (Run OT2842).

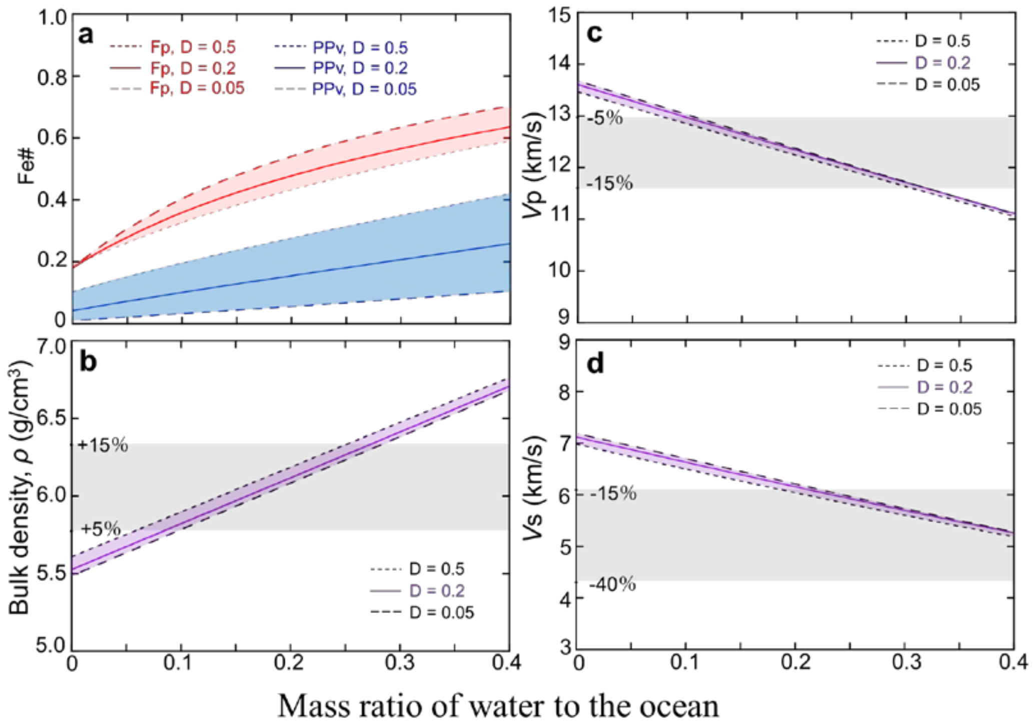

Supplementary Figure 4: Seismic velocities of FeO-rich layer as a function of water with different *D* values. *D* values ($(\text{Fe/Mg})_{ppv}$ / $(\text{Fe/Mg})_{fp}$) of 0.05 and 0.5 were used to explore the sensitivity of our results to variations in FeO partitioning**. a**. Changes in the composition (*Fe#*, Fe/(Mg+Fe)) of minerals. **b–d,** Change in density (ρ), V_p, and V_s. Shaded areas indicate the changes in ρ (+5–+15 %), V_p (−5–−15 %), and V_s (−10–−40%). The mass of the ocean corresponds to 1.38×10^{21} kg. PPv, post-perovskite; Fp, ferropericlase.

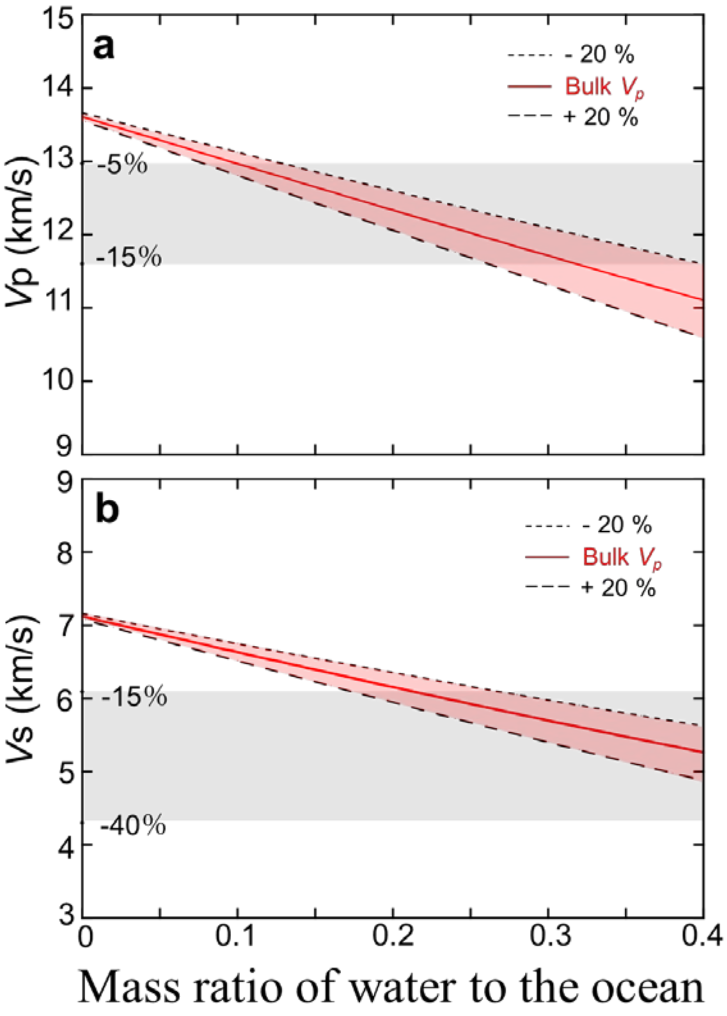

Supplementary Figure 5: Seismic velocities of FeO-rich layer considering their different composition dependence on ferropericlase. ±20% uncertainties of the composition dependence were tested. Shaded areas indicate the changes in ρ (+5–+15 %), V_p (−5–−15 %), and V_s (−10–−40%). The mass of the ocean corresponds to 1.38×10^{21} kg.

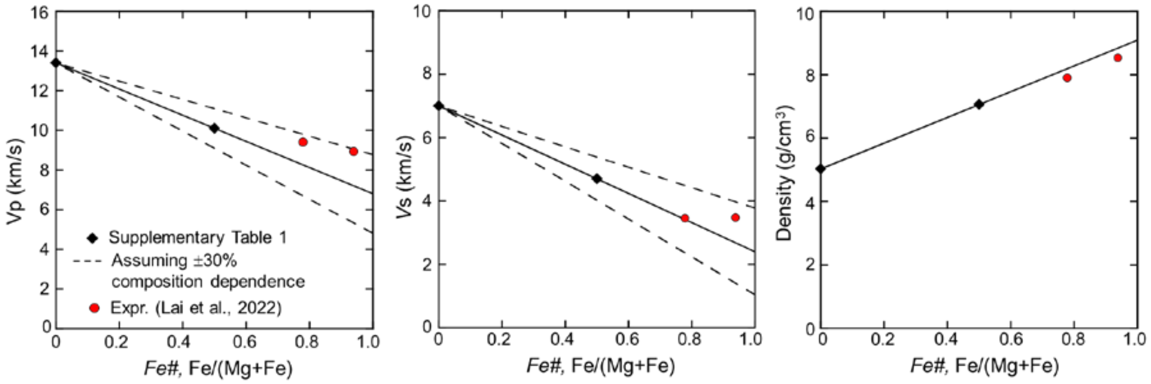

Supplementary Figure 6: Seismic velocities of ferropericlase considering their different composition dependence. ±30% uncertainties of the composition dependence were tested. This uncertainty reproduces the V_p and V_s obtained from experiments[8,9,57].

Supplementary Table 1. Experimental conditions and results.

Run No.	Capsule	Metal sample	Temperature (K)	Time (min)	Bridgmanite + H_2O (mm^3)	Contact Surface (mm^2)	Reaction Length (μm)
OT2842	MgO	Fe	2473	1	0.12	0.2	57 (6)
OT2933a	MgO	$Fe_{0.8}Ni_{0.2}$	2473	1	0.20	0.07	96 (5)
OT2933b	MgO	$Fe_{0.54}Ni_{0.46}$	2473	1	0.20	0.07	97 (11)
OT2782	MgO	Fe	2088	1	0.47	3.14	11 (6)
OT2844	MgO	Fe	1773	10	0.12	0.2	36 (8)
OT2829	MgO	Fe	1773	180	0.20	0.07	44 (9)
OT2830	MgO	Fe	1773	180	0.20	0.2	42 (13)
OT2780	MgO	Fe	1573	60	0.47	3.14	4 (3)
OT2915a	Au	Fe	1500	180	0	0.07	0
OT2915b	Au	Fe	1500	180	0.05	0.07	19 (2)
OT2915c	Au	Fe	1500	180	0.10	0.07	25 (5)
OT2934a	Au	Fe	1473	180	0.15	0.07	62 (4)
OT2934b	Au	$Fe_{0.8}Ni_{0.2}$	1473	180	0.05	0.07	52 (6)
OT2934c	Au	$Fe_{0.54}Ni_{0.46}$	1473	180	0.05	0.07	44 (6)

All experiments were conducted at 25 GPa.

Supplementary Table 2. Chemical composition of bridgmanite and ferropericlase in run products.

	Starting material	OT2829 [Solid Fe]	OT2933b [Liquid Fe-Ni]	OT2834b [Solid Fe-Ni]
bridgmanite				
Wt.%				
SiO_2	57.1 (1)	55.8 (21)	56.7 (7)	53.6 (43)
Al_2O_3	3.1 (5)	3.3 (13)	2.4 (2)	4.3 (23)
FeO		6.5 (25)	6.3 (22)	8.8 (59)
MgO	39.0 (6)	35.2 (18)	36.0 (19)	33.2 (29)
Total	99.2 (9)	96.5 (10)	101.5 (5)	99.8 (13)
O (fixed)	3	3	3	3
Si	0.98 (1)	0.97 (2)	0.97 (1)	0.94 (6)
Al	0.06 (1)	0.07 (3)	0.05 (1)	0.09 (5)
Fe		0.09 (4)	0.09 (3)	0.13 (9)
Mg	0.97 (1)	0.90 (3)	0.91 (3)	0.86 (5)
Fe/(Mg+Fe)	0	0.09	0.09	0.13
ferropericlase				
Wt.%				
SiO_2		0.5 (1)	1.2 (10)	1.3 (9)
Al_2O_3		-	0.8 (1)	-
FeO		63.4 (15)	41.3 (12)	79.4 (6)
MgO		34.5 (20)	52.2 (19)	15.1 (3)
NiO		-	5.4 (3)	5.0 (2)
Total		98.4 (10)	101.0 (5)	100.8 (10)
O (fixed)		1	1	1
Si		0.01 (1)	0.01 (1)	0.01 (1)
Al		-	0.01 (1)	-
Fe		0.50 (2)	0.29 (1)	0.70 (1)
Mg		0.49 (2)	0.65 (2)	0.23 (1)
Ni		-	0.04 (0)	0.04 (0)
Fe/(Mg+Fe)		0.51	0.31	0.75
$(Fe/Mg)_{ppv}$ / $(Fe/Mg)_{fp}$		0.10	0.22	0.05

Note that the minute amount of SiO_2 and Al_2O_3 components appearing in feropericlase are likely due to overlap with bridgmanite.

Supplementary Table 3. Parameters used for density, *V*p and *V*s calculations.

Parameter	composition		
Ferropericlase (Fp) Muir and Brodholt (2015)[6]		x = 1	x = 0.5
ρ (g/cm^3)	$(Mg_xFe_{1-x})O$	5.03	7.06
V_p (km/s)	$(Mg_xFe_{1-x})O$	13.4	10.1
V_s (km/s)	$(Mg_xFe_{1-x})O$	7.00	4.70
Bridgmanite (Brd) Zhang et al. (2016)[7]		x = 1	x = 0.75
ρ (g/cm^3)	$(Mg_xFe_{1-x})SiO_3$	5.33	5.72
V_p (km/s)	$(Mg_xFe_{1-x})SiO_3$	14.0	13.3
V_s (km/s)	$(Mg_xFe_{1-x})SiO_3$	7.20	6.50
Post perovskite (PPv) Zhang et al. (2016)[7]		x = 1	x = 0.75
ρ (g/cm^3)	$(Mg_xFe_{1-x})SiO_3$	5.40	5.80
V_p (km/s)	$(Mg_xFe_{1-x})SiO_3$	14.1	13.4
V_s (km/s)	$(Mg_xFe_{1-x})SiO_3$	7.5	6.8

All data represent values at 136 GPa and 4000 K.
